# Block Teeth

**Published:** 1862-11

**Authors:** 


### BLOCK TEETH.

By reference to our advertising sheet, it will be seen that our friend, Dr. Wardle, of this city, continues to manufacture block teeth and do everything in that line for the profession, and at a rate, of which certainly no one can complain. To those who have used his blocks and teeth, we need not say a word, and to those who have not, we can assure them that Dr. W. is not excelled, if equaled, by any other manufacturer of block teeth in the United States; they are excellent representatives of nature's work, in shade, form and arrangement. Those who become accustomed to mounting special block work usually very much dislike to mount the ordinary molded teeth and blocks, because of a want of adaptation, and the great amount of labor necessary to attain that object. Some of the finest sets of teeth we have ever seen in the mouth were from Dr. Wardle's hands. T.

